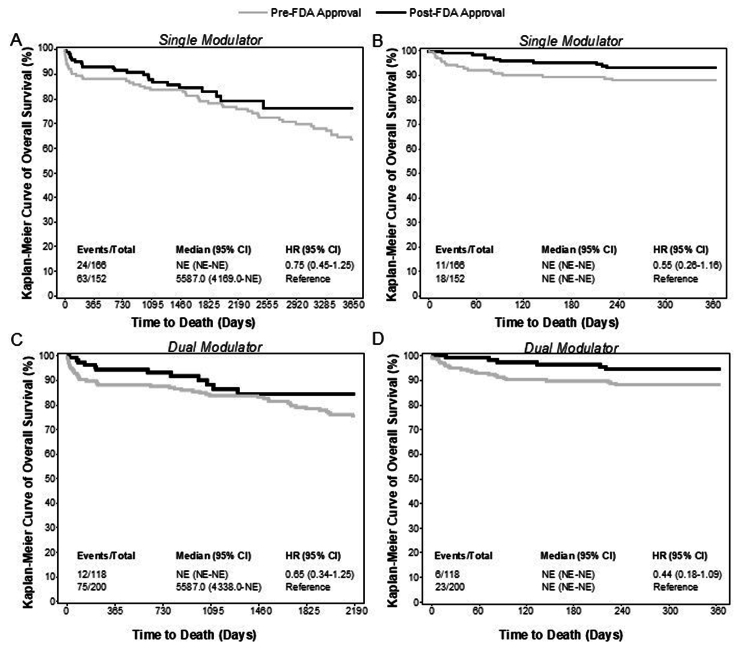# Impact of Cystic Fibrosis Transmembrane Conductance Regulator Modulating Therapies on Liver Transplant Outcomes

**DOI:** 10.1016/j.gastha.2025.100810

**Published:** 2025-09-14

**Authors:** Sara Naimimohasses, Ankit Ray, Eunice Tan, Asher Wiggins, Bima J. Hasjim, Shiyi Chen, Mamatha Bhat

**Affiliations:** 1 Ajmera Transplant Centre, University Health Network, University of Toronto, Toronto, Canada; 2 Department of Medicine, Yong Loo Lin School of Medicine, National University of Singapore, Singapore; 3 Department of Surgery, University of California – Irvine, Orange, California; 4 Transplant AI Initiative, Ajmera Transplant Centre, University Health Network, University of Toronto, Toronto, Ontario, Canada

**Keywords:** CFTR Modulators, Cystic Fibrosis-Related Liver Disease, Liver Transplant

## Abstract

**Background and Aims:**

Up to 40% of patients with cystic fibrosis (CF) develop CF-related liver disease (CFrLD), which can progress to the point of requiring liver transplantation (LT). Advances in CF transmembrane conductance regulator (CFTR) modulator therapies, especially triple therapy modulators, have significantly improved pulmonary outcomes, but their impact on LT for CFrLD remains unclear.

**Methods:**

Using data from the Scientific Registry of Transplant Recipients in 2000-2023, we analyzed trends in LT waitlisting for CFrLD pre- and post-U.S. Food and Drug Administration (FDA) approval of CFTR modulators: ivacaftor (January 31, 2012; single therapy), ivacaftor-lumacaftor (July 2, 2015; dual therapy), and ivacaftor-tezacaftor-elexacaftor (October 21, 2019; triple therapy). We compared the waitlist characteristics and post-LT outcomes of pre- and post-FDA approval eras.

**Results:**

Of 258,090 patients waitlisted for LT, 551 (0.2%) had CFrLD. The proportion of CFrLD patients on the LT waitlist decreased after FDA approval of triple CFTR modulators (0.23% to 0.14%; *P* < .0004). Patients waitlisted after FDA approval of single and dual CFTR modulators were, on average, older (17.4 vs 20.6 years; *P* < .001 and 18.0 vs 20.8 years; *P* = .004). Median model for end-stage liver disease-sodium scores were higher among individuals waitlisted following the approval of dual (9 [6–14] vs 10 [8–15], *P* < .013) and triple (9 [6–14] vs 12.5 [8–17], *P* < .003) CFTR modulators. There were no significant differences in post-LT survival pre- and post-FDA approval of single, dual, or triple CFTR therapy.

**Conclusion:**

These findings suggest that CFTR modulators may mitigate CFrLD complications and delay the need for waitlisting as physicians await the patient’s response to therapy and reassess the need for LT.

## Introduction

Cystic fibrosis (CF) is one of the most prevalent autosomal recessive disorders, affecting approximately 37,000 individuals across North America.^1^ Progressive lung disease due to recurrent and chronic infections is the chief cause of morbidity and mortality among individuals with CF.^2^ Additionally, defects in the CF transmembrane conductance regulator (CFTR) may lead to CF-related liver disease (CFrLD), the third leading cause of death among the CF population.^3^^,^^4^ Up to 40% of individuals with CF have some degree of CFrLD that ranges from abnormalities in liver biochemistry, steatosis due to CF-associated metabolic complications, and drug-induced liver injury.^5^ Furthermore, patients with CFrLD can develop noncirrhotic portal hypertension due to porto-sinusoidal vascular disease or focal biliary fibrosis.^5^^,^^6^ Approximately 10% of individuals with CFrLD will progress to cirrhosis and are at risk of developing hepatic decompensation complications necessitating liver transplantation (LT).^5^^,^^6^

CF is a monogenetic disorder caused by mutations in the CFTR gene, which encodes a chloride ion channel expressed on the surface of epithelial cells.^5^ Although there are over 2000 genetic variants, the phenylalanine residue deletion at position F508 on chromosome 7 is the most frequently occurring disease-causing mutation.^2^ Over the last decade, advances in sequencing the CFTR gene have driven the development of small molecule therapies called CFTR modulators, which work to correct the defects in the CFTR and restore its proper function.^7^ Ivacaftor, the first CFTR modulator approved by the U.S. Food and Drug Administration (FDA) in 2012, also known as a CFTR “potentiator,” improves the transport of chloride through the CFTRs by binding directly to the channel, helping to keep them open and thereby facilitate the transport of chloride. It is recommended for use in individuals with CF who have gating mutations, such as G551D, present in approximately 10% of the CF population.^1^^,^^7^^,^^8^ Subsequently, the dual CFTR modulator lumacaftor-ivacaftor was approved in 2015, with lumacaftor, a CFTR “corrector,” improving the processing of CFTR and its transport to the cell surface. Due to its design, this dual modulator is suitable for use in individuals homozygous for the F508 deletion, prevalent in 50% of individuals with CF.^1^^,^^9^ More recently, the triple CFTR modulator, ivacaftor-tezacaftor-elexacaftor was approved in 2019. Tezacaftor and elexacaftor function as CFTR correctors that work to improve the folding and presentation of mature CFTR to the cell membrane, with tezacaftor also acting as a potentiator, binding to CFTR at an alternate site to ivacaftor, and enhancing the function of CFTR.^10^ The triple CFTR modulator has the broadest applicability and is suitable for use in individuals with at least one F508 mutation, comprising 85.4% of individuals with CF.^1^^,^^11^

CFTR modulators have resulted in significant improvements in pulmonary function, reduced referrals for lung transplantation, and increased overall survival.^12^^,^^13^ However, the impact of these therapies on downstream outcomes of CFrLD (eg, LT waitlisting, rates, and outcomes) is currently limited to case series and remains to be explored.^14^ Thus, we sought to investigate the practice patterns of LT waitlist, characteristics, and post-LT outcomes for patients with CFrLD pre- and post-FDA approval of CFTR modulators from a large, national transplant registry in the United States, with a particular focus on the period following the introduction of triple therapy CFTR modulators.

## Methods

### Data Collection

This was a retrospective study from the Scientific Registry of Transplant Recipients (SRTR). The SRTR data system includes data on all donors, wait-listed candidates, and transplant recipients in the United States, submitted by the members of the Organ Procurement and Transplantation Network. The Health Resources and Services Administration, U.S. Department of Health and Human Services, provides oversight of the activities of the Organ Procurement and Transplantation Network and SRTR contractors. This study focused on patients waitlisted for LT and transplanted for CFrLD between January 31, 2000, and June 2, 2023. Data were analyzed for three distinct periods based on the FDA approval of CFTR modulators as a surrogate for their availability to treat CF-related complications: 1) single CFTR modulator therapy (ivacaftor; approved in January 31, 2012), 2) dual CFTR modulator therapy (ivacaftor-lumacaftor; approved in July 2, 2015), and 3) triple CFTR modulator therapy (ivacaftor-tezacaftor-elexacaftor; approved in October 21, 2019). Patients waitlisted for LT were stratified primarily into pre- (before October 21, 2019) and post-FDA (after October 21, 2019) approval of triple therapy. Additional results stratifying patients’ pre- and post-FDA approval of single and dual therapy were reported in the Supplemental Materials. Each modulator period includes patients waitlisted pre- and post-approval of the respective modulator. Due to overlapping approval timelines, some patients were included in both the pre- and post-periods of multiple eras of modulator approval.

### Variables of Interest

The primary outcome of interest was the proportion of patients waitlisted for LT, graft, and overall survival. Key demographic and clinical characteristics included age, sex, race/ethnicity, and other liver-related complications at the time of listing.

### Data Analysis

Descriptive statistics were performed to summarize the baseline characteristics of patients waitlisted for LT before and after each FDA approval. Categorical variables were presented as counts and proportions, while continuous variables were summarized as means (standard deviations [±SD]) or median (interquartile ranges [IQR]). Depending on the data distributions, differences in categorical variables between groups were assessed using chi-square tests or Fisher’s exact test, whereas two-sample *t*-tests or two-sample Wilcoxon tests were used for continuous variables. Complete case analyses were used for handling missing data, where records with missing data for one or more variables were excluded from the analyses.

The Kaplan-Meier method was used to estimate both 1-year post-LT survival and long-term post-LT survival among patients waitlisted in the pre- and post-approval periods for each of the three CFTR modulator therapies. Survival was defined as the time from waitlisting to death or last follow-up, with censoring at the last follow-up if applicable. Durations for long-term survival were measured from the date of therapy introduction until the end of the observation period (2022). Differences in survival between pre- and post-approval groups were tested using log-rank tests. Statistical analyses were conducted using SAS version 9.4 (SAS Institute, Cary, NC, USA), with a statistical significance threshold of *P* < .05.

## Results

### Patient Demographics

A total of 258,090 individuals were waitlisted for LT during the review period. Of these, 551 (0.2%) were waitlisted for CFrLD as their primary indication for LT. Patient demographics are summarized in Table 1. The majority of patients were male (57.9% male vs 42.1% female), 96.4% were White, 2.4% were Black, 0.9% were multiracial, and 0.4% were Asian.

**Table 1:** Demographic Details of Patients Waitlisted for LT With CFrLD as the Primary Indication

	Total (*N* = 551)
Candidate age at listing (y)	
Mean (SD)	18.97 (10.06)
MELD-Na at listing	
Median (IQR)	10.0 (7.0–14.0)
Sex^a^	
Female	232 (42.11%)
Male	319 (57.89%)
Blood type^a^	
A	212 (38.48%)
B	42 (7.62%)
AB	10 (1.81%)
O	287 (52.09%)
Race^a^	
Asian	2 (0.36%)
Black	13 (2.36%)
White	531 (96.37%)
Multi	5 (0.91%)
Donor type^a^	
Deceased	278 (87.42%)
Living	40 (12.58%)
Comorbidities: diabetes, dialysis, and chronic obstructive pulmonary disease status at listing^a^	
Diabetes	25 (25.25%)
Hemodialysis	4 (1.30%)
Chronic obstructive pulmonary disease	24 (8.33%)

a Values are expressed as absolute numbers with percentages expressed in parentheses. Percentages were calculated after accounting for missingness.

**Figure 1:**
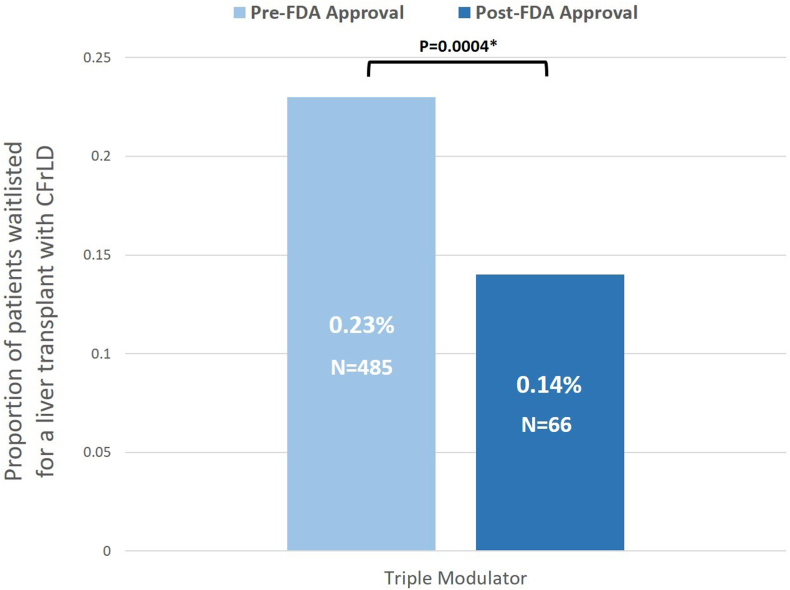
Alterations in the proportion of individuals waitlisted for LT pre- and post-FDA approval of triple CFTR modulators (ivacaftor-elexacaftor-tezacaftor).

**Figure A1:**
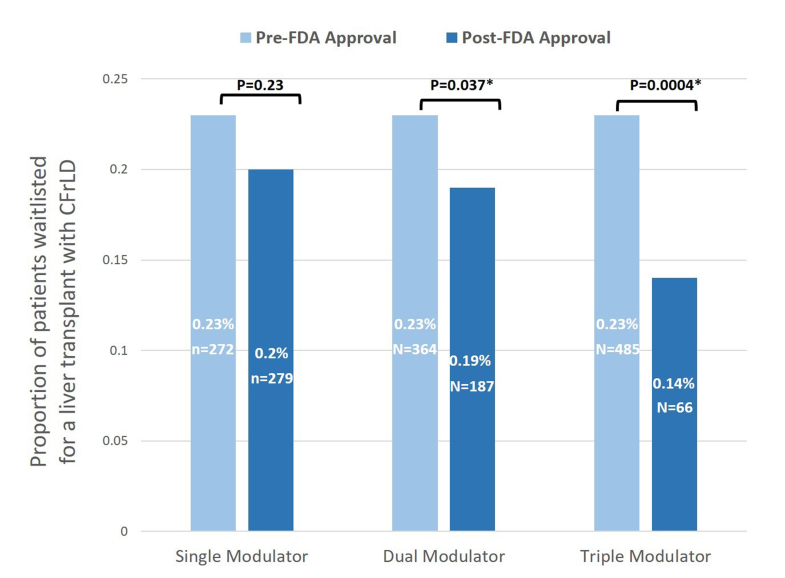


Comparisons were made between the proportion of individuals waitlisted for LT across the CFTR modulator FDA approval periods (Figure A1). There were lower rates of waitlisting for LT after FDA approval of the triple CFTR modulator, ivacaftor-tezacaftor-elexacaftor (0.23% post-FDA approval vs 0.14% pre-FDA approval, *P* = .0004; Figure 1).

### Clinical Characteristics of Waitlisted Patients

Patients waitlisted after FDA approval of the single and dual CFTR modulators were, on average, older (17.4 ± 9.12 vs 20.6 ± 10.68 years, *P* < .001 and 18.0 ± 9.34 vs 20.8 ± 11.13 years, *P* = .004) (Table A1). This trend was observed pre- and post-approval of the triple CFTR modulator; however, it did not achieve statistical significance (18.6 ± 9.62 vs 21.7 ± 12.58 years, *P* = .06). Furthermore, a significant increase in the median model for end-stage liver disease-sodium (MELD-Na) scores was observed with the approval of dual (9 [6–14] vs 10 [8–15], *P* < .013) and triple (9 [6–14] vs 12.5 [8–17], *P* < .003) CFTR modulators (Table 2).

**Table 2:** Comparison of Liver Transplant Candidate Characteristics and Outcomes Pre- and Post-FDA Approval of Triple CFTR Modulators (Ivacaftor-Elexacaftor-Tezacaftor)

	Pre-triple modulator (January 31, 2000-October 21, 2019) (*N* = 485)	Post-triple modulator (October 21, 2019-June 2, 2023) (*N* = 66)	*P* value
Candidate age at listing (y), mean (±SD)	18.6 ± 9.62	21.7 ± 12.58	.06
Model for End-Stage Liver Disease-Sodium at listing, median (IQR)	9 (6–14)	12.5 (8–17)	.003
Bilirubin at listing (mg/dL), median (IQR)	1.1 (0.6–2.7)	1.7 (1–3.4)	.012
Removed from waitlist due to clinical improvement, N (%)	40 (19.4%)	0 (0%)	.13
Developed graft failure post-transplant, N (%)	28 (10.1%)	1 (2.4%)	.15

### Waitlist Delisting Due to Clinical Improvement

There were no significant changes in the delisting rates pre- and post-approval of triple CFTR modulators (Table 2). There was a significant increase in bilirubin in the triple CFTR modulator cohort (1.1 [0.6–2.7] vs 1.7 [1–3.4], *P* = .012).

### Waitlist and Post-Transplant Survival

**Figure 2:**
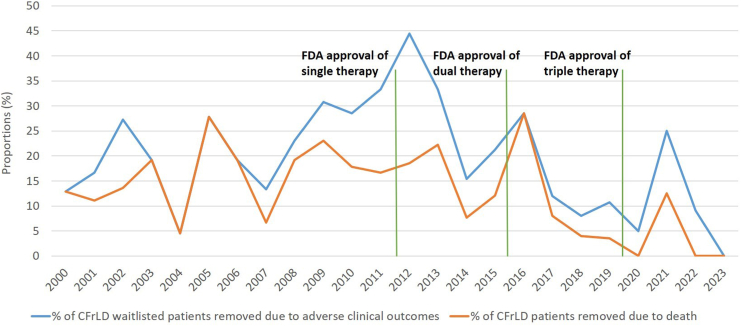
Annual proportion of CFrLD patients removed from liver transplant waitlist solely due to death or any adverse clinical outcome.

Given the pathophysiology of CF and its well-established pulmonary involvement, our next step was to compare the causes of death among patients waitlisted and those successfully transplanted, focusing on rates of infection and respiratory failure. Following undifferentiated “other” causes of death, respiratory failure, and/or acute respiratory distress syndrome remained the primary cause of death among individuals who were waitlisted for CFrLD (Table 3). There were no significant differences in causes of death between pre- and post-approval eras of other CFTR modulator therapies (Table A2). Similarly, when reviewing causes of death among individuals who were successfully transplanted, there were no significant differences between pre- and post-FDA approval dates (Table A3). Trends in the proportion of CFrLD patients removed from the waitlist stratified by cause (solely due to death or all adverse clinical outcomes, such as health deterioration or medical unsuitability for transplant) were also analyzed (Figure 2).

**Table 3:** Causes of Death/Dropout Among Individuals Waitlisted and Transplanted for CFrLD Pre- and Post-FDA Approval of Triple CFTR Modulators (Ivacaftor-Elexacaftor-Tezacaftor)

Population	Cause of death	Pre-approval triple modulator (January 31, 2000-October 21, 2019)^a^	Post-approval triple modulator (October 21, 2019-June 2, 2023)^a^
Waitlisted	Cardiovascular	3 (4.8%)	1 (33.3%)
	Malignancy	1 (1.6%)	0
	Other causes	37 (59.7%)	1 (33.3%)
	Renal failure	1 (1.6%)	0
	Respiratory failure/ARDS	10 (16.1%)	1 (33.3%)
	Infections	10 (16.1%)	0
	Total	62	3
Transplanted	Cardiovascular	13 (15.3%)	0
	Graft failure	3 (3.5%)	0
	Malignancy	1 (1.2%)	0
	Other causes	37 (43.5%)	2 (100%)
	Renal failure	1 (1.2%)	0
	Respiratory failure/ARDS	22 (25.9%)	0
	Infections	8 (9.4%)	0
	Total	85	2

ARDS, acute respiratory distress syndrome.

a Values are expressed as absolute numbers, with percentages calculated relative to the total number of individuals in each time period.

**Figure 3:**
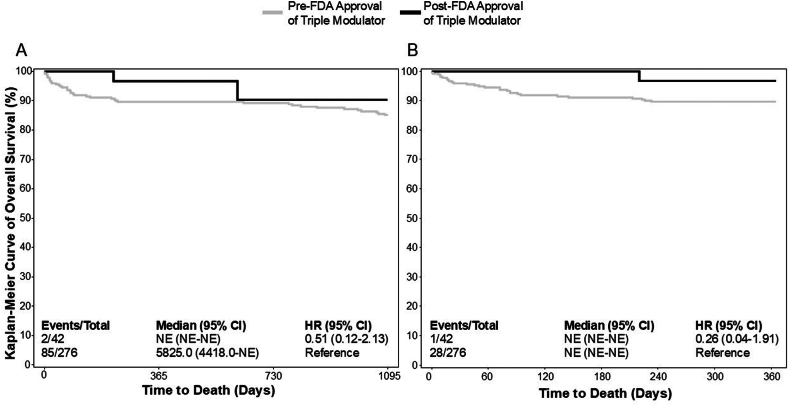
Long-term survival following LT pre- and post-FDA approval of triple CFTR modulators (ivacaftor-elexacaftor-tezacaftor), (A) 3-year post-liver transplant survival prior to and following FDA approval of ivacaftor-tezacaftor-elexacaftor (October 21, 2019; log-rank *P* value = .3447). (B) 1-year post-liver transplant survival prior to and following FDA approval of ivacaftor-tezacaftor-elexacaftor (log-rank *P* value = .1537).

Lastly, we analyzed long-term survival following LT. There were no statistically significant differences in 1- and 10-year survival pre- and post-FDA approval of the single CFTR modulator ivacaftor nor 1- and 6-year survival pre- and post-FDA approval of the dual CFTR modulator lumacaftor-ivacaftor (Figure A2). Additionally, there were no significant differences in 1- and 3-year post-LT survival pre- and post-FDA approval of the triple CFTR modulator (Figure 3).

## Discussion

CFrLD is a rare indication for LT, and the reported impact of CFTR modulators on the liver in the literature remains largely restricted to case series.^14^ In the present study, we found that rates of waitlisting for CFrLD decreased over time and were lower after FDA approval of triple therapy CFTR modulators. Additionally, patients in the post-FDA approval era had higher MELD-Na scores at waitlisting, suggesting delayed waitlisting to observe patient trajectories on modulator therapy. Once transplanted, there were similar long-term outcomes pre- and post-FDA approval. These findings suggest that CFTR modulators may be associated with a stabilizing effect on the progression of severe CFrLD and could potentially improve LT-free outcomes. Ongoing development of precision medicine therapies in CFTR and other rare diseases is essential for optimizing pre- and post-LT outcomes.

Triple CFTR modulators have been previously found to have a profound reduction in waitlist additions in lung transplantation.^13^ Similarly, we found a modest decrease in LT waitlisting for CFrLD after FDA approval of triple CFTR modulators. Prior clinical trials on CFTR modulators have yielded mixed results regarding their effects on the liver. During phase 3 clinical trials and in clinical practice, CFTR modulators have been associated with increased liver enzymes.^11^^,^^15^ These nonspecific effects were also observed in small cohorts of LT recipients with CFTR modulators.^11^^,^^16^^,^^17^ However, the mechanisms behind these elevated liver enzymes are not fully understood. Conversely, Tachtatzis *et al* biopsied four individuals with CF on CFTR modulator therapy and found their biopsies were histologically normal with no indication of drug-induced liver injury.^17^ This evidence supports both the safety and efficacy of CFTR modulators in post-transplant patients within the setting of elevated liver enzymes. Even less is known about the effect of these agents on the progression of CFrLD. In a retrospective, single-center study of 84 pediatric CF patients, Levitte *et al.* observed significant reductions in liver enzymes, including gamma-glutamyl transferase, and in biomarkers of hepatic fibrosis, such as the aspartate aminotransferase-to-platelet ratio index and the gamma-glutamyl transferase-to-platelet ratio, among patients with liver involvement who were treated with dual CFTR modulator, ivacaftor-lumacaftor.^18^ However, these biomarkers did not significantly improve in those treated with the triple CFTR modulator, ivacaftor-tezacaftor-elexacaftor suggesting there may be variability in its effects on CFrLD depending on specific modulators employed, warranting further exploration.^18^

Assessments of CFTR modulator impact on liver stiffness, measured by vibration-controlled transient elastography, present conflicting results. In a prospective study by Calvo *et al.*, consisting of 55 CF patients on triple CFTR modulator therapy, triple modulators showed a significant reduction in liver stiffness after six months, indicating a potential role for these therapies in attenuating CFrLD.^19^ By comparison, Tewkesbury et al. reported no significant change in fibrosis markers across 74 CFTR modulator-treated patients after a median of 21 months. Notably, patients with elevated baseline liver stiffness (>6.8 kPa) showed a marked reduction over time (−3.3 kPa vs 0.25 kPa, *P* < .01), while those with initially normal liver stiffness did not exhibit significant changes.^20^ This suggests that CFTR modulators may offer the greatest benefit for patients with more advanced hepatic fibrosis, while their impact may be less pronounced in those with milder disease. However, these agents may still provide a stabilizing effect, as no progression was reported in patients with minimal baseline fibrosis. Furthermore, a retrospective review by Ramsey *et al* of over 7000 adult CF patients reported reduced rates of progression to cirrhosis in those treated with single and dual CFTR modulators.^21^ In our study, a higher MELD-Na score observed among CFrLD patients in the post-approval eras of therapies may reflect an increased capacity for recovery among younger individuals with less severe liver disease. Collectively, these findings highlight the potential of CFTR modulators to improve the trajectory of CFrLD.

CFTR modulators act systemically, correcting defective CFTR expressed on epithelial cells but also on apical cholangiocytes within the liver. The downstream effects of CFTR modulators in the liver environment are not known, but there are multiple hypotheses. Returning the CFTR function leads to increased chloride ion release into the bile fluid, which causes the release of bicarbonate into the bile duct through a chloride/bicarbonate exchanger, AE2.^22^ This may theoretically help to delay the progression of CFrLD by enhancing biliary bicarbonate secretion, forming a “bicarbonate umbrella” protecting cholangiocytes from the toxic effects of bile acids.^22^ Additionally, enhanced bicarbonate secretion increases biliary osmotic pressure, drawing more water, thereby reducing the viscosity and enhancing biliary flow.^22^ While this hypothesis is sound in theory, clinical evidence fails to support it as inspissated bile is rarely a reported finding in CFrLD patients.^23^^,^^24^ Changes in biliary composition may also have beneficial downstream impacts on the intestinal microbiome, though further research is required.^23^

Our study has the following limitations. First, as our data were gathered retrospectively, we cannot establish definitive causal relationships between the introduction of specific CFTR modulators and changes in LT rates. Second, we used FDA approval dates to define distinct CFTR modulator therapy eras, which may not fully capture clinical practice variations. Adoption of new therapies likely varied across centers, with some patients remaining on older modulators despite the availability of newer ones. Importantly, due to our reliance on retrospective registry data, we cannot confirm or report the exact proportion of patients actively receiving CFTR modulators or the specific types of modulators administered in each time period. It is also important to note that advancements in the management of CF and CFrLD have occurred in parallel with the introduction of CFTR modulators, which include improved nutritional support, antibiotic use, pulmonary care, recognition and management of liver disease, and developments in immunosuppressant protocols.^4^ Currently, the SRTR does not capture these factors in granular detail, though future collaborations with the Cystic Fibrosis Foundation Patient Registry may provide further insights into CFrLD.^25^ Furthermore, CFTR modulators have been associated with reductions in hepatic fat deposition,^26^ which may contribute to improved liver-related outcomes. Nevertheless, our study encompasses a large, national cohort of LT candidates with CFTR and provides valuable insights into a rare but relevant cause of liver disease.

Collectively, these findings underscore the complexity of CFTR modulators' effects on CFrLD, although they appear to have some benefit, as CFrLD patients waitlisted for LT after the approval of dual and triple CFTR modulators were overall older and had a higher MELD-Na score. Future research highlighting the effects of CFTR modulators on other CFrLD-specific endpoints (eg, fibrosis) is warranted and may ultimately optimize CF outcomes in both the pre- and post-transplant settings.

## Figures and Tables

**Figure A2:**