# Supplementary material for: Impact of Cystic Fibrosis Transmembrane Conductance Regulator Modulating Therapies on Liver Transplant Outcomes
Source: Gastro Hep Adv. 2025 Sep 14;5(2):100810. doi: 10.1016/j.gastha.2025.100810 (PMC12681715; doi:10.1016/j.gastha.2025.100810)
Supplement: Supplementary Materials [file mmc1.docx]

**Table 1:** Comparison of liver transplant candidate characteristics and outcomes pre- and post-FDA Approval of CFTR Modulators (Ivacaftor, Lumacaftor-Ivacaftor, and Ivacaftor-Elexacaftor-Tezacaftor)

|  | Pre-single modulator  (Jan 31^st^ 2000- Jan-31^st^ 2012) | Post-single modulator  (Jan 31^st^ 2012 -June 2^nd^ 2023) | Pre-dual modulator  (Jan 31^st^ 2000- July2^nd^ 2015) | Post-dual modulator  (July 2^nd^ 2015-June 2^nd^ 2023) |
| --- | --- | --- | --- | --- |
| **N** | 272 | 279 | 364 | 187 |
| **Candidate Age at Listing (years)*** | 17.4±9.12 | 20.6±10.68 | 18.0±9.34 | 20.8±11.13 |
| **P value** | <0.001 | | 0.004 | |
| **MELD at listing*** | 9 (6-14) | 10 (8-15) | 9 (6-14) | 10 (8-15) |
| **P value** | <0.001 | | 0.013 | |
| **Bilirubin at listing* (mg/dL)** | 1.1 (0.7 – 2.7) | 1.1 (0.7 – 2.7) | 1.1 (0.7 – 2.7) | 1.2 (0.7 – 2.7) |
| **P value** | 0.37 | | 0.36 | |
| **Number de-waitlisted due to clinical improvement** | 13  13.1% | 27  22.7% | 29  20% | 11  17.8% |
| **P value** | 0.07 | | 0.71 | |
| **% developed graft failure post-transplant** | 17  11.2% | 12  7.2% | 22  11% | 7  5.9% |
| **P value** | 0.22 | | 0.13 | |

** Values expressed as mean±standard deviation or median (Interquartile range), P value reflects significant across each time period.*

**Table 2**: Causes of death/dropout amongst individuals waitlisted for CFrLD pre- and post-FDA approval of single and dual CFTR modulators

| Cause of death | Pre-approval  single modulator  (Jan 31^st^ 2000- Jan-31^st^ 2012) | Post-approval single modulator  (Jan 31^st^ 2012 -June 2^nd^ 2023) | Pre-approval  dual modulator  (Jan 31^st^ 2000- July2^nd^ 2015) | Post-approval dual modulator  (July 2^nd^ 2015-June 2^nd^ 2023) |
| --- | --- | --- | --- | --- |
| Cardiovascular | 3  (9.7%) | 1  (2.9%) | 1  (2.0%) | 3  (18.8%) |
| Malignancy | 1  (2.9%) | 0 | 1  (2.0%) | 0 |
| Other causes | 22  (64.7%) | 16  (51.6%) | 28  (57.1%) | 10  (62.5%) |
| Renal failure | 0 | 1  (3.2%) | 1  (2.0%) | 0 |
| Respiratory failure/ARDS | 5  (14.7%) | 6  (19.4%) | 8  (16.3%) | 3  (18.8%) |
| Infections | 5  (14.7%) | 5  (16.1%) | 10  (20.4%) | 0 |
| Total | 34  (52.3%) | 31  (47.7%) | 49  (75.4%) | 16  (24.6%) |

** Values expressed as absolute numbers with percentages expressed in parentheses*

**Table 3**: Causes of death amongst individuals transplanted for CFrLD pre- and post-FDA approval of single and dual CFTR modulators

| Cause of Death | Pre-approval  single modulator  (Jan 31^st^ 2000- Jan-31^st^ 2012) | Post-approval single modulator  (Jan 31^st^ 2012 -June 2^nd^ 2023) | Pre-approval  dual modulator  (Jan 31^st^ 2000- July2^nd^ 2015) | Post-approval dual modulator  (July 2^nd^ 2015-June 2^nd^ 2023) |
| --- | --- | --- | --- | --- |
| Cardiovascular | 7  (11.1%) | 6  (25.0%) | 8  (10.7%) | 5  (41.7%) |
| Graft failure | 1  (1.6%) | 2  (8.2%) | 2  (2.7%) | 1  (8.3%) |
| Malignancy | 1  (1.6%) | 0 | 1  (1.3%) | 0 |
| Other causes | 29  (46.0%) | 10  (41.7%) | 35  (46.7%) | 4  (33.3%) |
| Renal failure | 1  (1.6%) | 0 | 1  (1.3%) | 0 |
| Respiratory failure/ARDS | 17  (27.0%) | 5  (20.8%) | 21  (28.0%) | 1  (8.3%) |
| Infections | 7  (11.1%) | 1  (4.2%) | 7  (9.3%) | 1  (8.3%) |
| Total | 63  (72.4%) | 24  (27.59%) | 75  (86.2%) | 12  (13.8%) |

** Values expressed as absolute numbers with percentages expressed in parentheses*
